# Artificial intelligence-based dairy cattle behavior recognition for estrus detection via ensemble fusion of two camera views

**DOI:** 10.1371/journal.pone.0340999

**Published:** 2026-01-16

**Authors:** Panawit Hanpinitsak, Tatpong Katanyukul, Norrawit Tonmitr, Chanon Suntra, Sora-at Tanusilp, Arthit Phuphaphud

**Affiliations:** 1 Department of Computer Engineering, Faculty of Engineering, Khon Kaen University, Khon Kaen, Thailand; 2 Department of Electrical Engineering, Faculty of Engineering, Khon Kaen University, Khon Kaen, Thailand; 3 Department of Animal Science, Faculty of Agriculture, Khon Kaen University, Khon Kaen, Thailand; 4 Department of Agricultural Engineering, Faculty of Engineering, Khon Kaen University, Khon Kaen, Thailand; UPR: University of the Poonch Rawalakot, PAKISTAN

## Abstract

Monitoring cattle behavior plays an important role in improving farm productivity, maintaining animal welfare, and supporting efficient management practices. This study presents a multi-view behavior recognition system that uses synchronized top-view and front-view CCTV footage, combined with deep learning techniques. The system includes four main components: cow identification, behavior classification, identity-behavior association using Intersection-over-Union (IoU), and a decision-level ensemble to combine information from both views. YOLOv8 models are applied separately to each camera angle to detect individual cows and classify six key behaviors: drinking, eating, standing, lying, riding, and chin resting, with the latter two being relevant for estrus detection. The system matches cow identities to their behaviors within each view and then integrates the results to produce a final activity label for each cow.

## Introduction

Monitoring cattle behavior is important for managing herd health, improving productivity, and detecting estrus in dairy farms. Key behaviors like eating, drinking, riding, and chin resting provide useful indicators for cows’ health and estrus condition. However, these behaviors are often tracked manually by farmers, which is time-consuming and prone to error, especially on large farms [1].

To overcome these challenges, various monitoring tools have been introduced. Wearable sensors offer one solution but can be uncomfortable for the animals, which impacts their well-being [2]. In contrast, computer vision using CCTV cameras provides a non-invasive and scalable alternative. Traditional image processing methods such as [3,4] can detect obvious and sudden movements like riding but often miss subtle behaviors and are sensitive to environmental changes.

Recent work has explored the use of artificial intelligence (AI) and machine learning to infer behavioral and welfare-related states in livestock, highlighting the potential of automated approaches to support animal welfare and farm management without extensive manual monitoring [5,6]. Building on this broader progress in AI, deep learning using convolutional neural networks (CNNs) have improved the robustness of vision-based behavior recognition by learning features directly from data. Previous studies have demonstrated CNN-based detection of various behaviors, including riding [7–9], lying [10], feeding [11], lameness [12], and rumination [13]. However, most models rely on single-camera setups, which limits detection in crowded scenes and often focus on a narrow range of behaviors.

Some recent efforts have aimed to expand behavior categories and improve accuracy using more advanced deep learning models like Long Short-Term Memory (LSTM), 3D-CNN, and attention mechanisms [14–16]. While effective, these approaches often involve high computational complexity and still face challenges related to occlusion. One multi-view study [17] attempted to address this issue by using re-identification techniques across camera views, but it did not cover certain key estrus behaviors like riding and focused more on extending coverage than improving classification performance. In addition, to the best of the authors’ knowledge, no prior studies have pointed out that certain behaviors may be inherently difficult to observe from particular camera angles, and leveraging the complementary strengths of different viewpoints may resolve such angle-specific limitations.

To address these limitations, we propose a relatively lightweight modular system using synchronized top and front CCTV views. Each camera is processed with YOLOv8 to detect individual cows and classify six behaviors. An IoU-based module links identities to behaviors, and a decision-level ensemble combines outputs from both views to resolve the conflict in case the behavior detection from front view and top view cameras are different. This approach offers a simple, effective solution for real-time behavior monitoring.

Therefore, the novelty of this work is that we are the first, to our knowledge, to propose a lightweight multi-view behavior recognition framework that explicitly leverages complementary strengths of synchronized top and front camera views to mitigate angle-specific misclassifications. Therefore, our method directly improves behavior classification accuracy for estrus detection, covering critical estrus-related behaviors such as riding and chin-resting that are overlooked in [17].

## Methodology

All procedures involving animals in this study were reviewed and approved by the Institutional Animal Care and Use Committee (IACUC) of Khon Kaen University, Thailand, under approval number IACUC-KKU-128/66, dated 19 October 2023. The committee reviewed the study in accordance with the Ethical Principles of Animal Experimentation of the National Research Council of Thailand. In addition, written consent to install CCTV cameras and conduct behavioral observation on cattle was obtained from the Dean of the Faculty of Agriculture, Khon Kaen University, who oversees the university dairy farm where the study was conducted.

This section describes a modular pipeline for detecting cow identities and behaviors using synchronized CCTV footage from top and front views. It consists of four main modules: (i) behavior detection, (ii) cow identification, (iii) IoU-based association, and (iv) ensemble fusion, as shown. Each module is explained in detail in the following subsections.

### Behavior detection and cow identity recognition

For each camera angle, two YOLOv8 models were independently trained: One for behavior classification and another for cow identity recognition. The YOLOv8 model was selected based on the real-time detection capability. The behavior model identifies six key activities related to estrus: standing, lying, drinking, eating, chin-resting, and riding.

Both models were trained with the same hyperparameter configuration: 300 epochs, batch size of 32, an image resolution of 640×640 pixels, and the Adam optimizer [18] with a learning rate of 0.001. To improve detection robustness in varying lighting conditions especially at night, for cow identity models, each image is augmented twice by applying the grayscale with the probability of 25%, saturation with the range of -25% to 25%, and blur up to 2.5px.

### IoU module and ensemble fusion module

#### IoU module.

**Fig 1:**
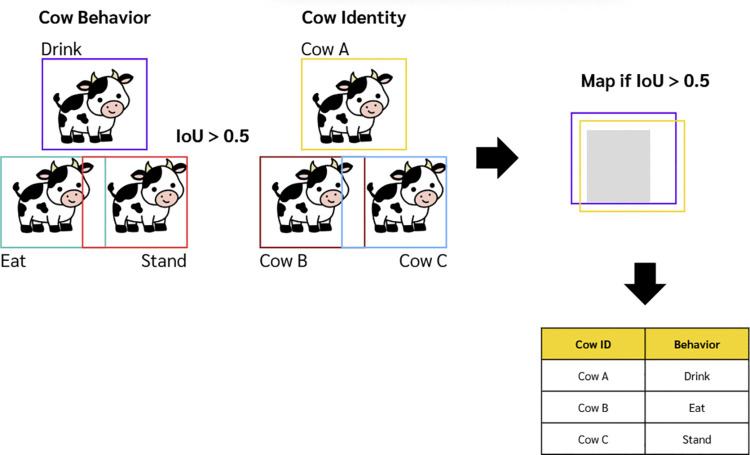
IoU module concept illustration.

Fig 1 depicts the concept of IoU module. Once the two YOLOv8 models (cow ID and behavior) produce bounding boxes for each frame, the IoU module calculates the Intersection over Union (IoU) between cow ID and behavior bounding boxes. Each cow ID is then matched with the behavior based on comparing the IoU value with the predefined IoU threshold. If the IoU between any pair of cow ID and behavior bounding boxes is greater than the threshold, that cow ID is mapped to that behavior. If none of the IoU values for a given cow ID exceed the threshold, the behavior is considered uncertain in the current frame, and the algorithm assigns the behavior from the previous frame. Similarly, if a cow is not detected at all by the identity recognition model, the algorithm also assigns it to the behavior from the previous frame. These fallback mechanisms ensure continuity in behavior tracking, even when detection is uncertain or missing.

#### Ensemble fusion module.

**Fig 2:**
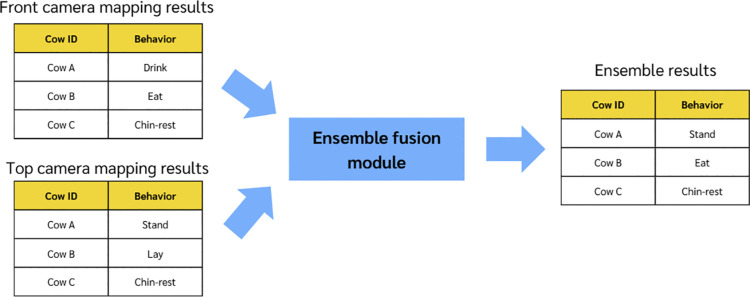
Ensemble module concept illustration.

Fig 2 illustrates the concept of ensemble fusion module. After the IoU module is separately applied to the top and front camera views, the mapping result (which cow performs which behavior) of each view will be obtained. However, occasionally the behavior obtained from the front and top views are different. For this purpose, the ensemble module is proposed to integrate the predictions, which resolve the detection discrepancies. The ensemble module is designed based on the empirical analysis of both the front and top view detection compared with the ground truth.

Although the front-view model generally performs better overall, certain misclassifications are observed in specific scenarios. Specifically, two common misclassification errors are as follows:

1. **False drinking detection**: Cows merely standing or walking near the water trough are often misclassified as “drink” due to their proximity, despite exhibiting no drinking behavior.
2. **False chin-resting detection**: When cows align closely in the pen, especially in rear-to-front configurations, the model occasionally detects a “chin-rest” or “ride” due to alignment-induced occlusion, despite the cow simply standing.

To address these issues, a simple rule-based fusion strategy is applied. If the top-view predicts “stand” while the front-view predicts “drink” or “chin-rest,” the top-view result is preferred to avoid common front-view errors. In other cases, the front-view prediction is used. This improves accuracy by leveraging the strengths of both views.

## Data collection and preprocessing

### Measurement environment setup

**Fig 3:**
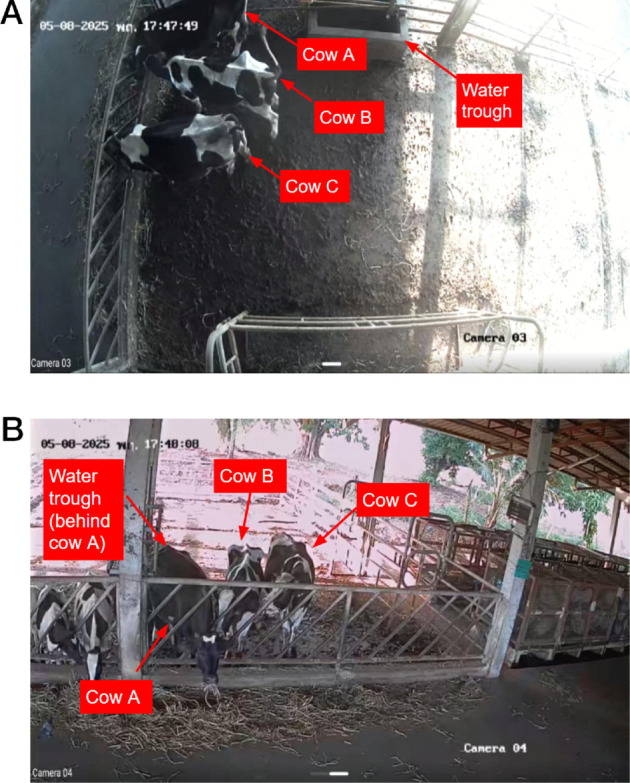
Comparison of different camera angles. (a) Top view camera angle, (b) Front view camera angle.

The experiment was conducted at the cattle farm of the Department of Agriculture, Khon Kaen University (KKU). Two fixed Hikvision CCTV cameras were installed to capture front and top views, each recording at 570 × 960 resolution and 10 frames per second. As shown in Fig 3, three 3-year-old heifers were placed in a 4 × 8 meter pen. For reference, they are labeled as cow A (black), cow B (white-dominant stripes), and cow C (black-dominant stripes).

### Image dataset for behavior and identity detection

To prepare the datasets for behavior and identity detection, video frames were extracted and cropped to 576 × 576 pixels. Bounding boxes and labels were added using Roboflow [19]. For each camera angle, two datasets were created: one for classifying behaviors (standing, lying, eating, drinking, chin resting, and riding) and another for identifying individual cows (cow A, B, and C). Table 1 shows the number of labels for each class. These labeled datasets allow the model to learn both behavior recognition and cow identification. The data were then split into training, validation, and test sets and exported as text files containing bounding box coordinates. After training, the model on the epoch that yielded the best validation mAP is used for the subsequent modules.

**Table 1:** Number of labels for each class.

Model	Class	Top view	Front view
Behavior Detection	Standing	872	1467
	Laying	337	282
	Eating	454	427
	Drinking	256	344
	Chin resting	260	351
	Riding	274	208
Cow Identity Detection	Cow A	229	151
	Cow B	235	149
	Cow C	224	151

### Video dataset for final system evaluation

To assess the system’s performance in a realistic setting, the complete behavior recognition pipeline (behavior detection, identity recognition, IoU matching, and ensemble fusion modules) was evaluated on a continuous video recording spanning 17 hours and 16 minutes (from 06:44:00 to 23:59:59). This evaluation was conducted on a day when two out of the three cows in the pen were confirmed to be in estrus, allowing the system’s ability to detect estrus-related behaviors under natural conditions to be assessed.

## Results and discussion

**Fig 4:**
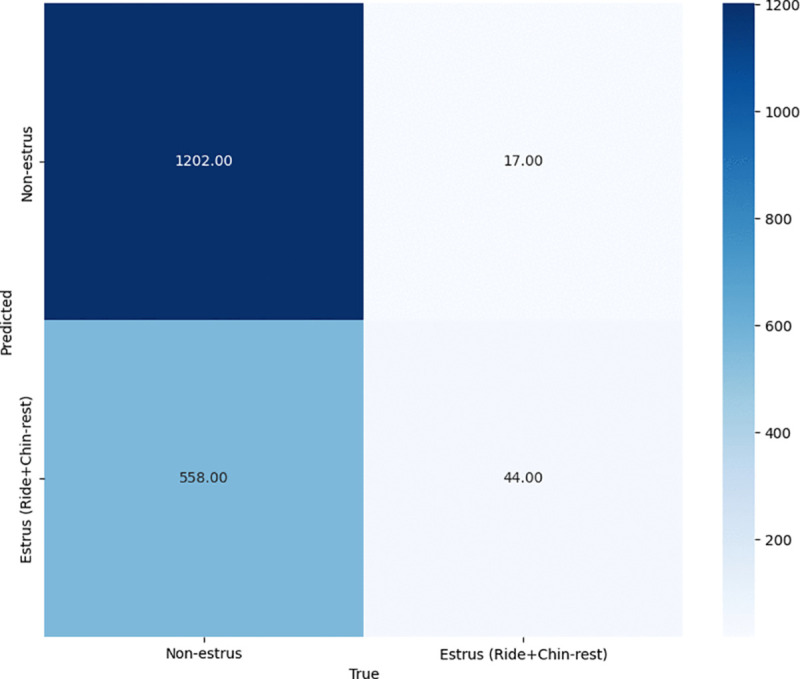
Confusion matrix of top view model predictions.

**Fig 5:**
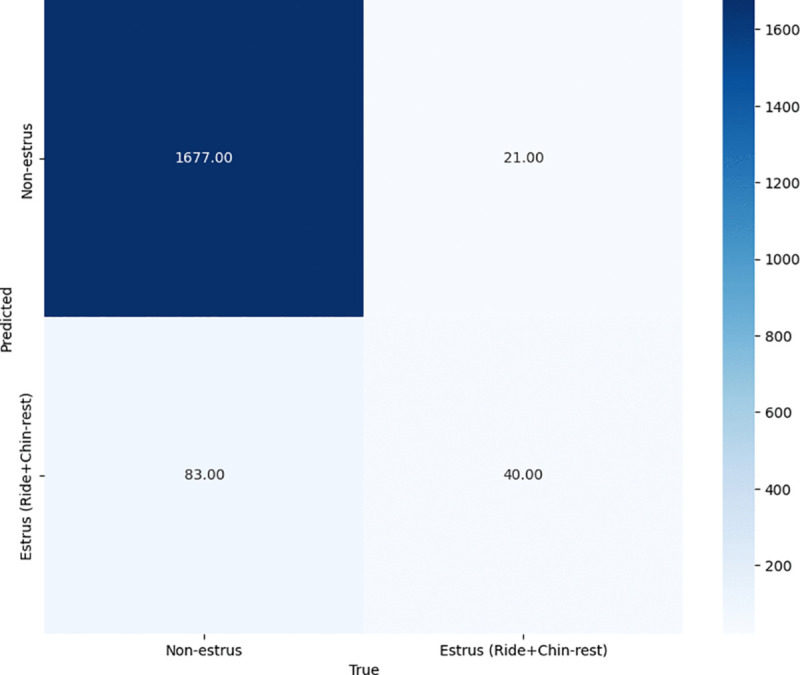
Confusion matrix of front view model predictions.

**Fig 6:**
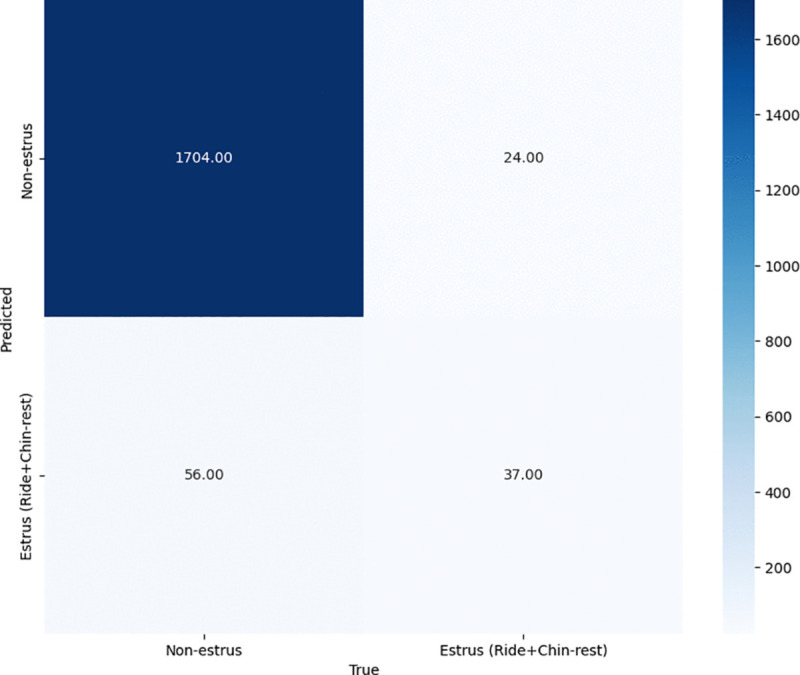
Confusion matrix of the proposed ensemble of both front and top views.

To evaluate the effectiveness of the proposed method in terms of estrus detection, we calculate the confusion matrix between the estrus-related behaviors (riding and chin-resting), and non-estrus-related behaviors (standing, laying, eating, drinking) on the video dataset as explained in the previous section. Figs 4–6 show the results of the recognition performance across three configurations: using only the top-view camera, only the front-view camera, and the proposed ensemble of both views. Additionally, Table 2 summarizes the F1-scores for each configuration.

**Table 2:** Performance comparison of behavior recognition using single and dual-view input.

Camera view	F1-score
Top view only	0.133
Front view only	0.435
Two-view ensemble	0.481

The top-view camera (Fig 4) demonstrates strong performance in identifying non-estrus behavior, with 1,202 true negatives. However, it struggles to detect estrus behavior, yielding only 44 true positives against 558 false positives, resulting in a low F1-score of 0.133. Most of these false positives stem from “standing” behavior being misclassified as “riding” or “chin-resting,” likely due to the limited ability of the top-down perspective to capture subtle postural cues.

In contrast, the front-view camera (Fig 5) shows improved performance across both classes. It achieves 40 true positives and 83 false positives for estrus behavior. The majority of these false positives are caused by “standing” behavior being misclassified as “chin-resting,” mainly due to alignment-induced occlusion when cows are closely positioned. Nonetheless, the front-view camera achieves a higher F1-score of 0.435, owing to its significantly lower false positive rate compared to the top view. This highlights the front view’s advantage in capturing posture-specific behaviors due to its more informative perspective.

The proposed two-view ensemble method (Fig 6) further improves the balance between precision and recall. It achieves 37 true positives and 56 false positives while maintaining a low false negative count. Notably, many instances that were incorrectly classified as “chin-resting” by the front-view model were correctly reclassified as “standing” when the top-view predicted “standing,” significantly reducing the false positive rate. Similarly, several “drinking” misclassifications by the front-view model were corrected by the top-view predictions. As a result, the F1-scores for “chin-resting” and “drinking” improved by approximately 4–5% compared to the front-view camera alone. The ensemble method ultimately yields the highest F1-score of 0.481, outperforming both individual views. These findings demonstrate that combining predictions from both views effectively compensates for the limitations of each camera, resulting in more accurate and robust detection of both estrus and non-estrus behaviors.

Overall, the results confirm that while the front view alone provides reasonable performance, integrating both views through a simple decision-level ensemble significantly enhances behavior recognition. The ensemble approach effectively mitigates common misclassifications—such as standing near a trough being mistaken for drinking or misidentifying chin-resting during occlusion, which improves the system’s reliability. However, the overall F1-score (0.481) remains low. This may be due to residual false positives in estrus-related behaviors and the high class imbalance in the dataset. Addressing this imbalance through resampling, class weighting, or loss function adjustments is left as future work.

## Conclusion and future works

In this paper, we proposed a multi-view cattle behavior recognition framework based on deep learning techniques, which is designed to operate with synchronized top-view and front-view CCTV footage. The system employs separate YOLOv8 models for cow identity recognition and behavior classification, integrated through an Intersection-over-Union (IoU)-based association module and a decision-level ensemble fusion module across views.

Experimental results demonstrate that the ensemble of both views outperforms single-view models, achieving the highest F1-score of 0.481 and correcting common misclassifications such as chin-resting and drinking. The findings confirm that integrating multiple camera perspectives helps overcome the limitations of individual views and enhances system robustness. This system may serve as a practical tool for dairy farmers by integrating it with existing herd management systems to provide early warnings for estrus, reduce labor burden, and improve breeding efficiency.

However, this study has several limitations. First, the F1-score of the final system remains relatively low at 0.481. A likely explanation is the significant class imbalance in the dataset, as shown in Table 1, which may cause the model to focus disproportionately on non-estrus behaviors that occur more frequently than estrus-related behaviors. Second, the ensemble fusion strategy used in this work is based on simple rule-based logic, which may not scale well to more complex scenarios. Implementing more advanced logic or techniques, such as using the detection confidence scores or temporal modeling, may yield further improvements. Furthermore, extending the system to recognize more subtle or complex behaviors such as restlessness, would further support comprehensive cattle welfare monitoring. These limitations will be addressed in the future work.
