# Correction: Artificial intelligence-based dairy cattle behavior recognition for estrus detection via ensemble fusion of two camera views

**DOI:** 10.1371/journal.pone.0346364

**Published:** 2026-03-31

**Authors:** Panawit Hanpinitsak, Tatpong Katanyukul, Norrawit Tonmitr, Chanon Suntara, Sora-at Tanusilp, Arthit Phuphaphud

The fourth author’s name is spelled incorrectly. The correct name is: Chanon Suntara. The correct citation is: Hanpinitsak P, Katanyukul T, Tonmitr N, Suntara C, Tanusilp S-a, Phuphaphud A (2026) Artificial intelligence-based dairy cattle behavior recognition for estrus detection via ensemble fusion of two camera views. PLoS One 21(1): e0340999. https://doi.org/10.1371/journal.pone.0340999.
